# Study on the γ/γ′ Eutectic Inhomogeneity of a Novel 3rd Generation Nickel-Based Single-Crystal Superalloy Casting

**DOI:** 10.3390/ma18214872

**Published:** 2025-10-24

**Authors:** Xiaoshan Liu, Anping Long, Haijie Zhang, Dexin Ma, Min Song, Menghuai Wu, Jianzheng Guo

**Affiliations:** 1 State Key Laboratory of Powder Metallurgy, Central South University, Changsha 410083, China; 193302080@csu.edu.cn (X.L.); 193302073@csu.edu.cn (A.L.); madexin@csu.edu.cn (D.M.); msong@csu.edu.cn (M.S.); 2 Shenzhen Wedge Central South Research Institute Co., Ltd., Shenzhen 518045, China; 3 School of Mechanical and Materials Engineering, North China University of Technology, Beijing 100144, China; 4 Department of Metallurgy, University of Leoben, A-8700 Leoben, Austria; menghuai.wu@unileoben.ac.at

**Keywords:** nickel-based superalloys, single crystal, γ/γ’ eutectic structure, distribution patterns, simulation

## Abstract

In the manufacture of single-crystal blades for aero-engines, the problem of eutectic aggregation on the upper surface of the blades has long been restricting the casting performance improvement. To investigate this phenomenon, this paper employs a simplified blade-like shape casting and focuses a 3rd generation nickel-based single-crystal superalloy as the research material. A systematic analysis is conducted to elucidate the distribution of γ/γ’ eutectic during solidification. Experimental results show distinct spatial variations in γ/γ’ eutectic distribution. Pronounced eutectic aggregation is observed on the upper surface of the blade but with sparse eutectic dispersion‌ on the lower regions of the casting. Relatively uniform eutectic distribution‌ dominates the mid-section of the specimen. To unravel the underlying mechanisms, this paper utilized a ‌multiphase volume-averaged solidification model‌, developed in prior work, to numerically simulate the γ/γ’ eutectic evolution during directional solidification. This computational framework enabled a comprehensive ‌quantitative analysis‌ of spatial and temporal variations in the eutectic volume fraction along the solidification direction. The integration of experimental and modeling approaches provides critical insights into the interplay between thermal gradients, alloy composition, and microstructural heterogeneity.

## 1. Introduction

Nickel-based superalloys serve as indispensable materials for critical high-temperature components, including aero-engine turbine blades and industrial gas turbine hot-section assemblies, operating reliably in extreme thermal environments (650–1000 °C) due to their exceptional mechanical stability, superior oxidation resistance, and remarkable hot-corrosion durability [1,2,3]. While single-crystal components are predominantly manufactured using Bridgman directional solidification technology, the strategic alloying with refractory elements such as rhenium (Re) in advanced-generation superalloys exacerbates microsegregation phenomena. This compositional design promotes excessive γ/γ′ eutectic phase formation within interdendritic domains during solidification [4,5,6]. Characterized by elevated volume fractions (typically 8–15%) and coarse morphological features, these eutectic structures exhibit limited solubility during standard solution heat treatment cycles, resulting in persistent eutectic retention at localized regions [7,8,9]. Such microstructural heterogeneities not only compromise the crystallographic continuity essential for single-crystal integrity but also serve as preferential sites for crack nucleation, thereby severely degrading creep resistance and fatigue performance under thermomechanical loading conditions. These microstructural imperfections have consequently emerged as a critical technological barrier impeding the widespread implementation of next-generation single-crystal superalloys in demanding aerospace applications [10,11,12,13,14]. 

The morphology, spatial distribution, and volume fraction of γ/γ’ eutectic in relation to solidification conditions have been systematically elucidated in multiple studies [15,16,17,18,19,20,21]. Brewster et al. [15] investigated the formation mechanism of surface eutectic layers in the directional solidification of the typical nickel-based superalloy CMSX-10N. They proposed that lateral solidification shrinkage induces interdendritic melt backflow as a volume compensation driving force, transporting solute-enriched low-melting-point liquid phases to near-surface regions and ultimately forming continuous surface eutectic layers. Cao et al. [16,17] further demonstrated through comparative experiments that mold-alloy interface interactions play a negligible role in surface eutectic formation. Instead, the phenomenon originates from the extrusion of residual liquid phases between dendritic skeletons to free surfaces under contraction stresses during the final solidification stage. Notably, Ma et al. [18] challenged conventional understanding of lateral eutectic enrichment in their study of third-generation single-crystal superalloy blades and test bars. While no directional migration of γ/γ’ eutectic toward surfaces was observed in the transverse direction (perpendicular to the solidification direction), a pronounced gradient distribution emerged along the axial direction (solidification direction): the volume fraction of eutectic reached 12–15% in the upper regions of castings but decreased to 3–5% in lower regions, revealing the profound regulatory effects of solidification pathways on solute redistribution.

Building upon previous research, this study systematically investigates the distribution patterns of γ/γ’ eutectic structures in a plate casting of a 3rd generation nickel-based single-crystal superalloy. Experimental characterization revealed significant spatial heterogeneity in eutectic distribution across different regions: distinct eutectic aggregation occurs in the upper section of the casting, sparse distribution in the lower section, and relatively uniform dispersion in the intermediate region. To validate the mechanisms governing these distribution characteristics, experimental and computational approaches was employed. Designed experiments enabled systematic microstructural characterization of γ/γ’ eutectic distribution. Concurrently, numerical simulations coupling temperature fields calculated using ProCAST 2019.0 software with a previously developed multiphase volume-averaged solidification model [22] were implemented to predict eutectic evolution during directional solidification. The multiphase volume-average solidification model was previously developed by the current authors [23] and has been adopted to model the directional solidification process of the superalloys [22,24]. The coexisting phases are treated as interpenetrating continua. The general framework can resolve equiaxed grains, columnar dendrites and liquid melt; however, because our study targets directional solidification of single-crystal superalloys, where equiaxed grains are intentionally suppressed, the computations reduce to a two-phase liquid-columnar model. It should be stressed here that when the local temperature falls below the eutectic temperature, the remaining interdendritic liquid freezes as a eutectic. Both liquid and columnar phases are represented on a fixed grid by their volume fractions; conservation of mass, momentum (for the liquid), energy/enthalpy, and solute is solved in mixture/phase-averaged form. The solid skeleton (columnar trunks) is treated as stationary for momentum, while the liquid is the only hydrodynamic phase [25]. Latent heat release and solute partitioning couple back to temperature and flow. Columnar dendrite trunks are idealized as step-wise cylinders advancing unidirectionally along the imposed thermal gradient. A constant primary dendrite arm spacing (λ_1_, taken from as-solidified microstructure) is assumed over the time scale of interest. The columnar tip/front is advanced using an LGK-type relation (Lipton–Glicksman–Kurz) [26], so that front kinetics depend on local thermal–solutal conditions. Local thermodynamic equilibrium at the solid–liquid interface provides interfacial concentrations $c_{l}^{*}$ and $c_{s}^{*}$. The growth of the columnar dendrites is governed by the concentration difference between the concentration difference ($c_{l}^{*}$ − $c_{l}$) between the averaged thermodynamic equilibrium concentration at the interface ($c_{l}^{*}$) and the local volume-averaged concentration ($c_{l}$). The interdendritic flow experiences a Darcy-type drag from the dendrite network. The mushy-zone permeability follows an extended Kozeny–Carman law as a function of liquid fraction and dendrite morphology (λ_1_), which controls resistance to flow and hence macrosegregation [27]. Buoyancy is treated by the Boussinesq approximation. With the above closure, the model predicts (i) the columnar front position, mush thickness and permeability evolution; (ii) 3D liquid flow and flow–solidification interaction terms that locally accelerate or suppress growth, thereby amplifying or damping channel formation; and (iii) the eutectic accumulation during the solidification of the superalloy.

In this study, a series of directional solidification experiments were conducted on advanced single-crystal superalloys to systematically investigate the formation of heterogeneous γ/γ′ eutectic structures. The spatial distribution and volume fraction of γ/γ′ eutectics within the solidified samples were quantitatively characterized using high-resolution microscopy and image analysis. Based on these experimental observations, numerical simulations were subsequently performed using a two-phase columnar–liquid solidification model to elucidate the underlying mechanisms driving eutectic accumulation. Finally, by integrating experimental measurements with numerical predictions, this work provides theoretical insights into the governing mechanisms of eutectic segregation and establishes a scientific basis for optimizing directional solidification processing parameters, aiming to improve microstructural uniformity and casting quality in next-generation turbine blade superalloys.

## 2. Experimental Design and Methods

### 2.1. Experimental Design

**Figure 1:**
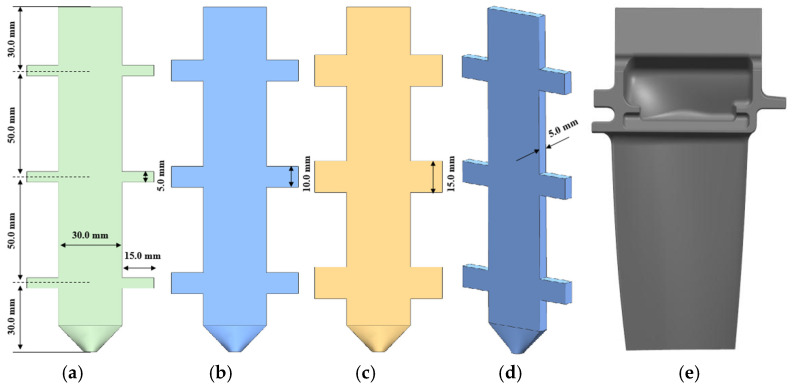
Geometrically graded plate castings with different platform features: (**a**) 5 mm height boss configuration; (**b**) 10 mm height boss configuration; (**c**) 15 mm height boss configuration; (**d**) cross-sectional thickness comparison; (**e**) representative of the single-crystal blade.

To systematically investigate the distribution characteristics of γ/γ’ eutectic content along the solidification direction in single-crystal castings, three different cross-section thickness plate castings with distinct geometric parameters were designed. The castings feature a rectangular base configuration (analogous to the aerofoil section of turbine blades) with a 30 mm central width and 5 mm uniform thickness. Symmetrical protruding platform structures (mimicking blade platforms or shrouds) were integrated on both sides to replicate the solidification characteristics of critical blade features. All platforms maintain a constant protrusion width of 15 mm, while their heights vary progressively at 5 mm, 10 mm, and 15 mm, establishing geometrically progressive solidification conditions. Figure 1 details the dimensional specifications of the three casting configurations.

### 2.2. Experimental Methods

The chemical composition of the investigated nickel-based single-crystal superalloy is listed in Table 1. Alumina-based ceramic shells were employed for mold preparation, with each shell accommodating 10 plate castings of varying dimensions to ensure experimental efficiency and data consistency. Directional solidification was conducted using a Bridgman furnace (ALD Vacuum Technologies GmbH, Hanau, Hesse, Germany) with precise thermal control.

**Figure 2:**
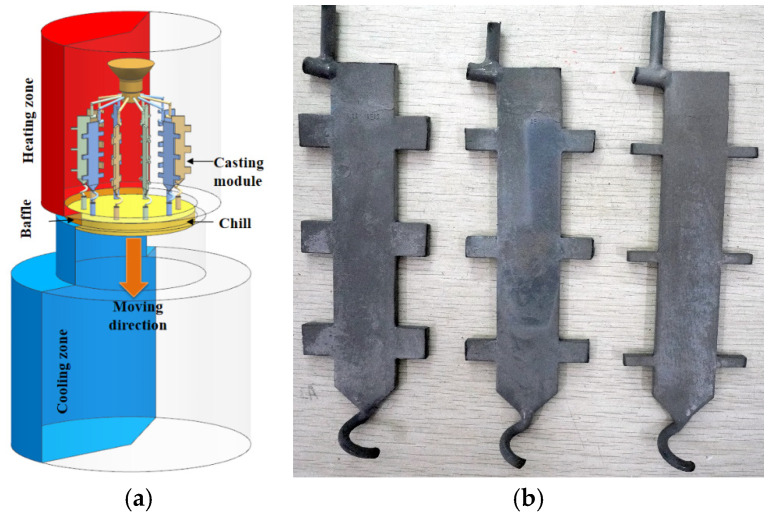
Bridgman furnace thermal zoning and as-cast specimens: (**a**) casting configuration; (**b**) three geometrically graded single-crystal plates.

The thermal zoning configuration of the Bridgman furnace and spatial arrangement of the mold assembly are detailed in Figure 2a. The single-crystal directional solidification process was carried out in a VIM-IC/DS/SC vacuum directional solidification furnace (ALD Vacuum Technologies GmbH, Hanau, Hesse, Germany). The specific steps of the casting process are as follows: Prior to pouring, the ceramic shell was preheated in the furnace insulation chamber at a temperature of 1793 ± 5 K. After the alloy was completely melted, the molten alloy, also at 1793 ± 5 K, was poured into the preheated shell. Subsequently, the pouring system was gradually moved from the high-temperature zone (1793 ± 5 K) to the low-temperature zone (353 ± 2 K) at a withdrawal rate of 3 mm/min to achieve directional solidification. A grain selector structure at the bottom of the casting ensured the stable formation of a single-crystal structure.

After the directional solidification process is completed, the ceramic shell was extracted from the furnace. After demolding, the pouring system was detached to obtain the final castings. As-cast specimens were then sectioned from the casting, mounted, ground, and polished. The polished surfaces were subsequently etched using a solution of CuSO_4_: HCl:H_2_O with a composition of 4 g:10 mL:20 mL. Metallographic examination was conducted using an MM-400 optical microscope (Olympus Corporation, Tokyo, Japan). The three types of single-crystal plate castings with different platform heights are shown in Figure 2b.

**Figure 3:**
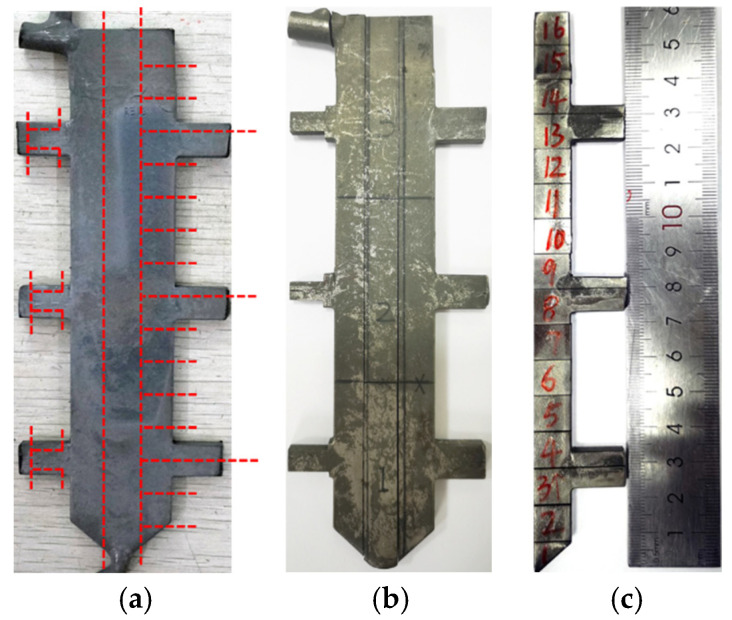
Specimen sampling protocol: (**a**) schematic sampling locations, (**b**) mid-span cross-section extraction, (**c**) prepared metallographic samples.

To systematically characterize the as-cast microstructural features, WEDM-DM300ZK wire electrical discharge machining machine (Suzhou SG Technology Co., Ltd., Suzhou, Jiangsu, China) was employed to extract metallographic specimens from three geometrically distinct plate castings following a predetermined sampling protocol. Figure 3 schematically details the standardized sampling locations across all casting configurations, ensuring positional consistency for comparative analysis. The metallographic specimens of the cut samples were meticulously ground, polished and etched. Finally, the metallographic structure images were obtained. The metallographic structures of the samples were photographed using a metallographic microscope. The metallographic images were processed and analyzed with the help of the Image-Pro Plus 6.0 image processing and analysis software to calculate the fraction of the γ/γ’ eutectic.

**Figure 4:**
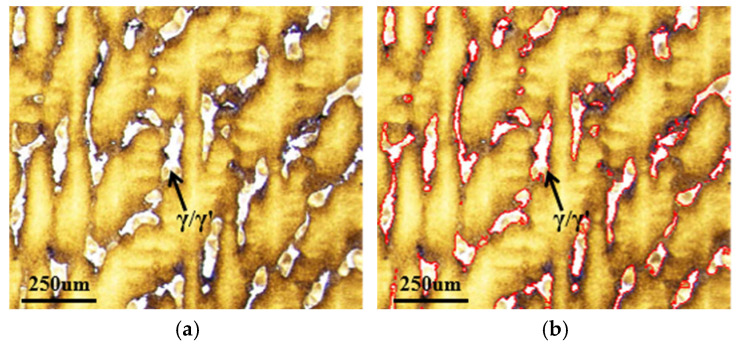
Measurement of the γ/γ’ eutectic structure area from the metallographic image using Image-Pro Plus software: (**a**) original metallographic micrograph; (**b**) resulting area measurement of the eutectic region.

Figure 4 illustrates the analysis of the distribution of γ/γ’ eutectic structures in metallographic images using Image-Pro Plus software. Specifically, Figure 4a displays the original metallographic image, while Figure 4b shows the corresponding marked eutectic regions. During measurement, all images subjected to analysis were uniformly processed with markings for γ/γ’ eutectic regions using the same RGB color threshold ranges [R value range (110, 255); G value range (110, 255); B value range (190, 255)] to ensure consistency throughout the procedure. The area of the marked eutectic regions in Figure 4b was then statistically quantified, and its percentage relative to the total image area was calculated to determine the eutectic area fraction.

## 3. Experimental Results and Analysis

### 3.1. Experimental Results

#### 3.1.1. Comparison of Eutectic Distribution on the Top and Bottom Surfaces of the Platform

**Figure 5:**
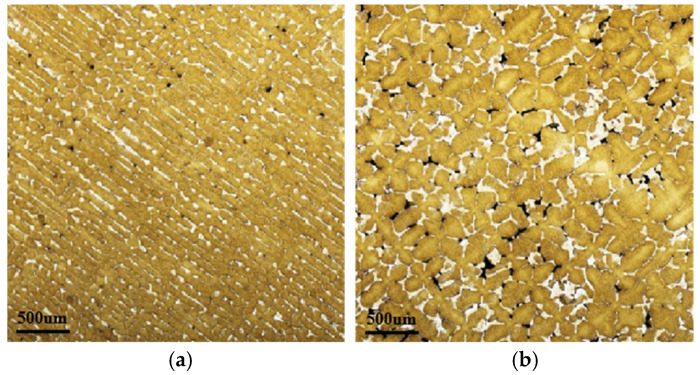
Metallographic images of as-cast microstructure at sampling positions of 10 mm height platform: (**a**) Bottom surface; (**b**) Top surface.

Figure 5 displays the as-cast microstructure of the bottom and top surfaces of the 10 mm platform. The brown dendritic structure corresponds to the γ matrix phase, while the white precipitates are the γ/γ’ eutectic. A significant difference in dendritic morphology is observed between the top and bottom surfaces. 

Figure 5a presents the as-cast metallographic image of the bottom surface of the 10 mm platform. As shown, the microstructure is predominantly composed of γ phase, with a low volume fraction of γ/γ’ eutectic structures. The dendrites are fine and exhibit a uniform size distribution. This morphology is attributed to the significant undercooling present at the bottom surface. As the solidification interface propagated upward, dendrites underwent rapid lateral growth, resulting in the formation of a refined transverse dendritic structure. Moreover, sufficient liquid feeding from the upper region prevented the formation of noticeable porosity defects in this area.

Figure 5b displays the as-cast metallographic image of the top surface of the 10 mm platform. It can be observed that the top surface contains a substantial amount of γ/γ’ eutectic structures. The dendritic sizes here are significantly larger than those at the bottom, indicating pronounced eutectic enrichment. Furthermore, owing to inadequate liquid feeding during solidification, relatively severe surface porosity occurred in this region, manifested as dark areas within the interdendritic spaces in the micrograph.

#### 3.1.2. Distribution of γ/γ’ Eutectic Along the Height Direction of Platforms

**Figure 6:**
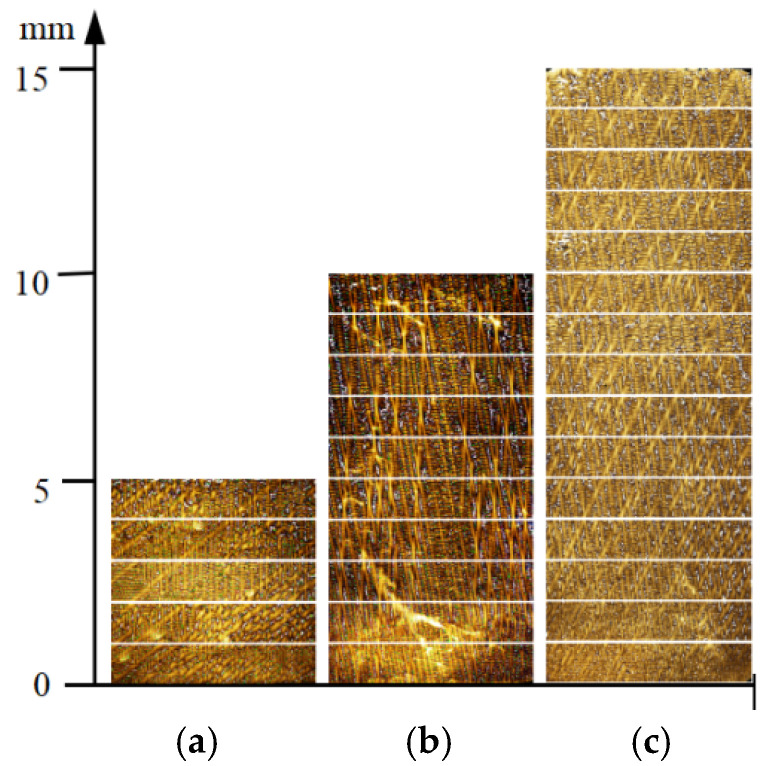
Metallographic images of as-cast microstructure along the solidification direction at sampling positions of three differently sized platforms: (**a**) 5 mm; (**b**) 10 mm; (**c**) 15 mm.

Figure 6 presents the as-cast microstructures along the solidification direction for three different height platforms (5 mm, 10 mm, and 15 mm). An in-depth analysis of the metallographic images was conducted to systematically reveal the evolution of the γ/γ′ eutectic along the solidification direction. To investigate the evolution of γ/γ’ eutectic along the solidification direction, the metallographic images of these three bosses were analyzed in detail. Each sample was divided into equal segments at 1 mm intervals along the height direction. The method described in Section 2.2 was applied to process the metallographic images of each 1 mm segment and quantify the γ/γ’ eutectic content.

**Figure 7:**
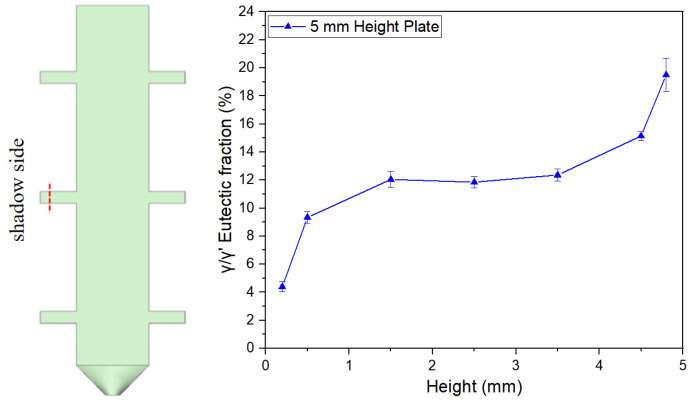
γ/γ’ eutectic content along the solidification direction of the 5 mm height plate.

**Figure 8:**
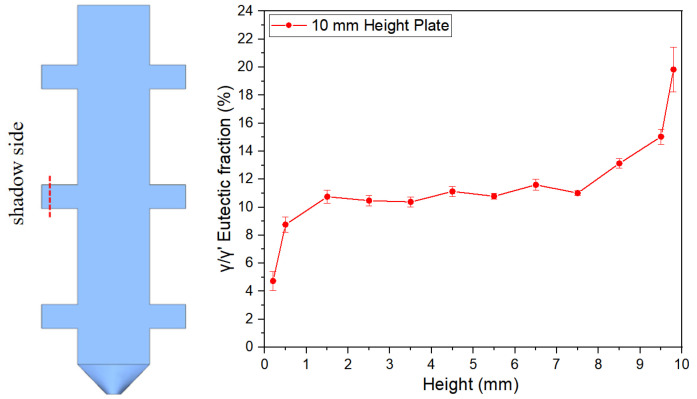
γ/γ’ eutectic content along the solidification direction of the 10 mm height plate.

**Figure 9:**
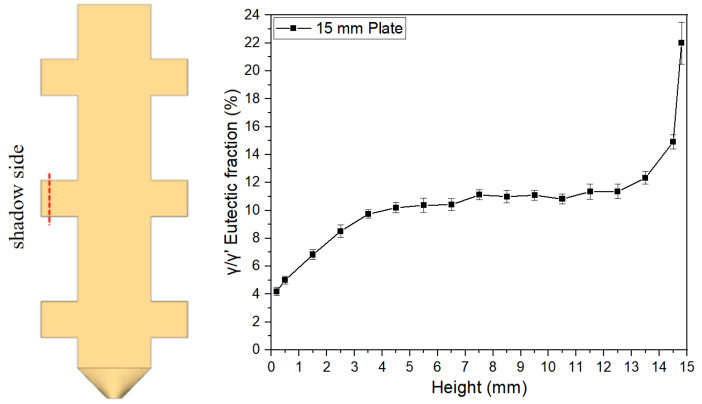
γ/γ’ eutectic content along the solidification direction of the 15 mm height plate.

Figure 7, Figure 8 and Figure 9 illustrate the variation trends of γ/γ’ eutectic content in the as-cast microstructure along the solidification direction at the sampling positions of the 5 mm, 10 mm, and 15 mm plates, respectively. Specifically, Figure 7 corresponds to the 5 mm height plate, Figure 8 to the 10 mm height plate, and Figure 9 to the 15 mm height plate. The curves in these figures graphically demonstrate the evolution of eutectic content during solidification at different plate heights, based on quantitative data. These results provide critical insights for subsequent analysis.

**Figure 10:**
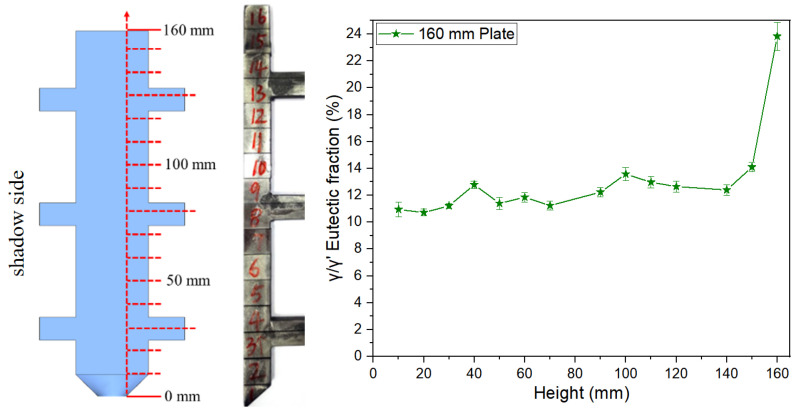
γ/γ’ eutectic content along the solidification direction at the transverse section sampling position of the 10 mm height plate.

To investigate the eutectic distribution characteristics along the height direction of the casting, this study employed 10 mm height plate castings as research objects. A transverse sectioning method was adopted to extract serial specimens perpendicular to the solidification direction, with quantitative metallographic analysis performed to determine the average γ/γ’ eutectic content at each height plane. As shown in Figure 10, the experimental data clearly reveal the nonlinear evolution of γ/γ’ eutectic content with solidification height.

#### 3.1.3. Eutectic Distribution at Corner Regions of the 10 mm Boss-Plate Junction

**Figure 11:**
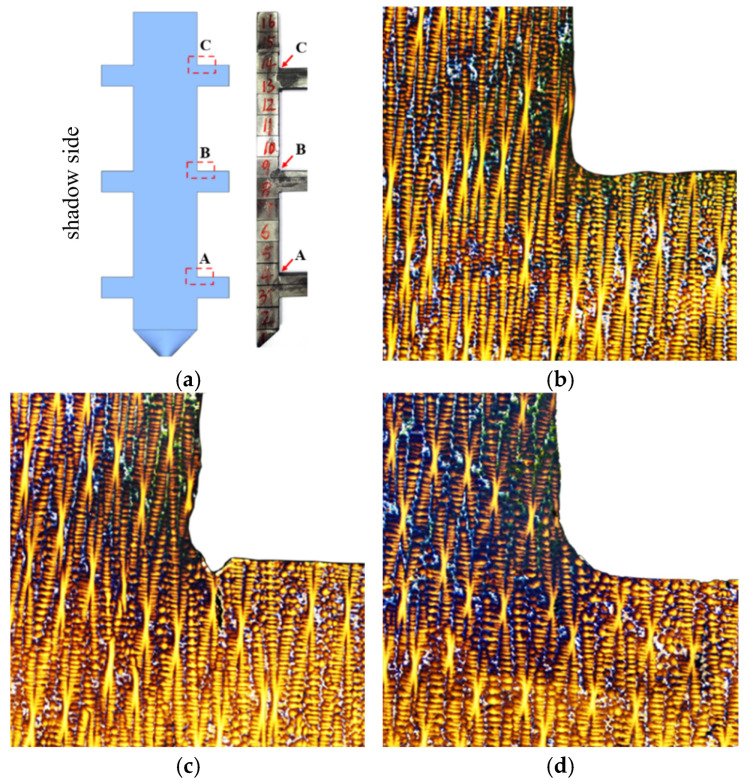
Metallographic images of sampling positions at three corners of the 10 mm height plate junction: (**a**) metallographic sampling positions, (**b**) metallographic image of Area A, (**c**) metallographic image of Area B, (**d**) metallographic image of Area C.

Figure 11 displays metallographic images of solidification microstructural features at corner regions of the 10 mm boss-plate interface. As shown in Figure 11a, three designated sampling points were selected within the geometric transition zones of the plates, with their corresponding low-magnification metallographic microstructures presented in Figure 11b–d. Systematic analysis of these images reveals that the γ/γ’ eutectic content in the corners is significantly reduced compared to adjacent regions. During the solidification process, this phenomenon indicates that the corner regions may exhibit thermal flow distribution characteristics and solute transport behavior that are distinct from those in the surrounding regions.

### 3.2. Analysis of Experimental Results

#### 3.2.1. Interpretation of γ/γ’ Eutectic Distribution Patterns

**Figure 12:**
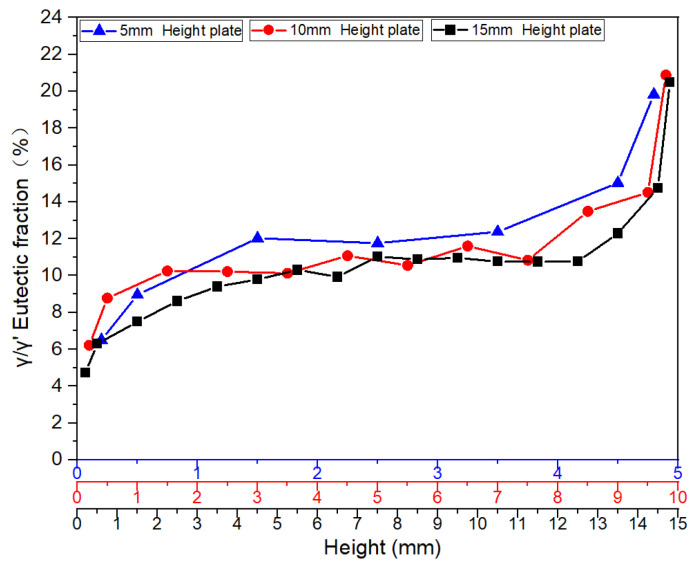
Comparison of γ/γ’ eutectic content variation curves in the as-cast microstructure along the solidification direction at sampling positions of three differently sized platforms.

Figure 12 illustrates the distribution characteristics of γ/γ’ eutectic content in the as-cast microstructure along the solidification direction for three differently sized plates (5 mm, 10 mm, and 15 mm). The results show that all specimens exhibit significant gradient variations in eutectic content as solidification progresses.

For the 5 mm plate, the γ/γ’ eutectic fraction at the bottom (6.51%) is significantly lower than that at the top (19.83%), with the top content being 3.05 times that of the bottom. This difference reflects a terminal enrichment phenomenon at the final solidification stage. For the 10 mm height plate, the eutectic contents at the bottom and top are measured as 6.22% and 20.88%, respectively, maintaining a consistent trend with the 5 mm specimen. Notably, in the 15 mm height plate, the bottom eutectic content drops to 4.74%, while the top remains as high as 20.50%, demonstrating an even steeper gradient. This distribution pattern may arise from the synergistic effects of dynamic solute redistribution at the solidification front and thermal gradients.

**Figure 13:**
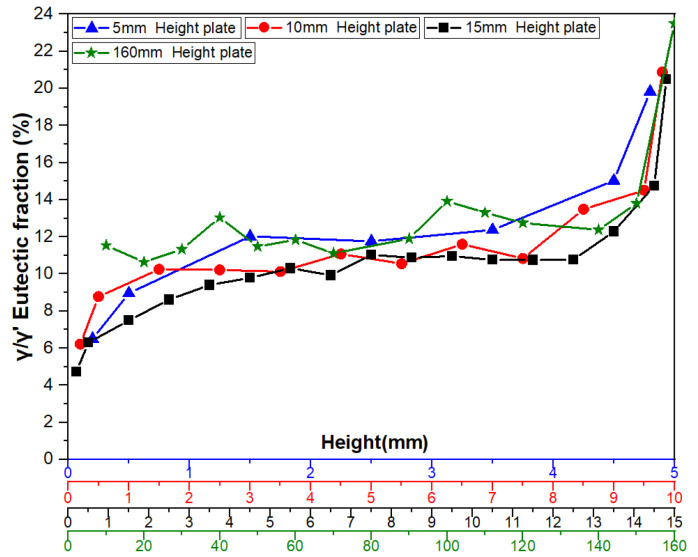
Comparison of γ/γ’ eutectic content variation curves in the as-cast microstructure along the solidification direction for different sampling positions.

Figure 13 further verifies the regularity of γ/γ’ eutectic formation and distribution during solidification by comparing variation curves across different sampling positions. A stable transition zone in eutectic content is observed in the mid-regions of all castings. Specifically, the average γ/γ’ eutectic fraction in the central region of the 5 mm boss is 11.76%. Corresponding values for the 10 mm and 15 mm bosses are 10.60% and 10.29%, respectively. For the 10 mm boss-plate casting, the central region exhibits an average eutectic fraction of 11.52% along its full height.

#### 3.2.2. Mechanism Discussion of γ/γ’ Eutectic Distribution Patterns

The findings above reveal the directional solidification characteristics of γ/γ’ eutectic in castings. Results in Figure 13 demonstrate that the top regions of each plate along the solidification direction exhibit significantly higher average size and volume fraction of γ/γ’ eutectic structures compared to the bottom regions, while the mid-regions display relatively uniform distributions. The spatiotemporal distribution of γ/γ’ eutectic structures is fundamentally a macroscopic manifestation of the dynamic equilibrium between solidification parameters (cooling rate, thermal gradients) and solute transport (diffusion, convection) [28,29,30]. Notably, in thick-section castings, prolonged melt persistence enhances long-range solute migration, leading to anomalous eutectic depletion at the bottom. This insight holds critical implications for optimizing directional solidification processes.

In the downward withdrawal directional solidification process (Figure 2a), the superalloy melt undergoes a counter-gravity solidification from bottom to top. In multicomponent alloy systems: high-density elements (e.g., Re, W) preferentially segregate to dendritic core regions; low-density elements (e.g., Al, Ti) accumulate in the interdendritic liquid phase.

As the solidification process proceeds, the solid fraction within the casting continues to rise, causing γ′-forming elements (such as Al and Ti) to be rejected into the remaining liquid phase, where they gradually accumulate. This eventually leads to the formation of γ/γ′ eutectic structures in the interdendritic residual liquid. Driven by the solute concentration gradient and assisted by melt convection, the enriched γ′-forming elements in the interdendritic liquid migrate upward along the solidification direction. Once the γ dendrites grow to the upper surface of the casting, further upward transport of these elements is hindered, resulting in their local enrichment in the top region. Consequently, the volume fraction of γ/γ′ eutectic increases markedly in the upper area, whereas the bottom region exhibits a decrease in eutectic content due to continuous solute loss. 

Remarkably, the uniform eutectic distribution in the central region likely arises from a dynamic balance among solute diffusion rates, melt convection intensity, and solidification rates. In this equilibrium state, the upward flux of Al/Ti aligns spatially with the advancing solidification front, establishing a stable solute distribution field that promotes eutectic uniformity.

This experimental finding shows excellent agreement with the planar front directional solidification theory established by M. Fleming in his seminal work, *Solidification Processing* [31]. Fleming systematically described the relationship between solute redistribution and interface stability during directional solidification, noting that under planar front growth conditions, the solidification interface continually rejects solute elements into the liquid phase, creating a solute concentration gradient along the solidification direction. Meanwhile, melt convection further enhances the transport of enriched solute to specific regions near the solidification front, ultimately causing localized accumulation at the end of solidification. The behaviors observed in this study—including the enrichment of γ′-forming elements (e.g., Al, Ti) in the interdendritic liquid, their upward migration along the solidification direction, and the notable increase in γ/γ′ eutectic volume fraction in the upper part of the casting—align well with the predictions of this theory. This agreement strongly demonstrates that Fleming’s solidification theory retains significant universal applicability and guiding value, even within multicomponent superalloy systems.

In the corner regions of the boss, the metallographic images in Figure 11 show a notably lower γ/γ′ eutectic fraction (5.0 ± 1.0%) compared with the adjacent plate regions (11.0 ± 1.0%). This difference arises from intensified shrinkage-induced flow during solidification. As the solidification front passes through the concave corner, the contraction of the cross-section drives solute-enriched liquid downward, causing solute depletion at the corner and enrichment in the adjacent expansion zone. The resulting solute deficiency leads to a reduced eutectic fraction in the corner region.

To elucidate the intrinsic physical mechanisms governing γ/γ’ eutectic distribution, this study will integrate numerical simulations to develop a multiphysics-coupled model incorporating temperature, solute, and flow fields. By simulating the spatiotemporal evolution of key parameters during directional solidification, the model will provide theoretical explanation and in-depth analysis of eutectic formation mechanisms.

## 4. Numerical Simulation of γ/γ’ Eutectic Evolution

### 4.1. Multiphysics-Coupled Modeling and Numerical Solution Strategy

#### 4.1.1. Computational Model

**Figure 14:**
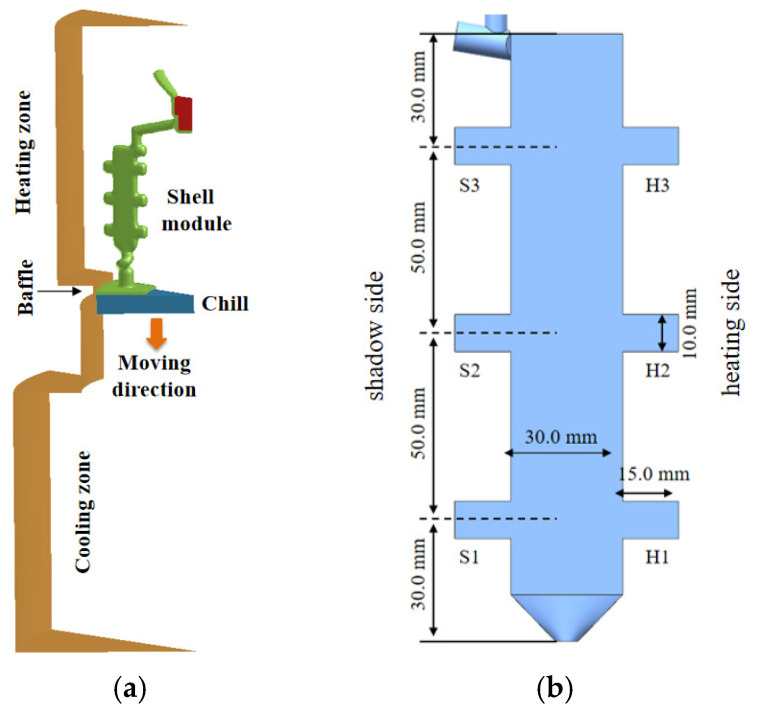
Temperature field simulation model: (**a**) Schematic of the module layout in the Bridgman furnace; (**b**) Dimensional details of the casting model.

Based on the physical essence of Bridgman directional solidification, a 3D transient multiphysics-coupled model was developed to analyze the synergistic evolution of temperature and solute fields in a 10 mm boss-plate casting (Figure 14a), as an example. To balance computational accuracy and efficiency, simulations were conducted using a 1/10th-scale geometric model.

Figure 14b details the dimensions of the 10 mm height plate casting. One narrow side of the casting is facing the heating wall (heating side), while the other side is back to the heating wall (shadow side). To investigate geometric effects on solidification behavior, particularly at abrupt cross-sectional transitions, three platforms were designated as H1, H2, H3 (heating side) and S1, S2, S3 (shadow side), each featuring 15.0 mm protrusions. This design simplifies the complexity of turbine blade geometries while retaining critical morphological features.

To simplify the simulation, the current multi-element superalloy is treated as eight Ni-X binary alloys with constant liquidus slope and solute partition coefficient. The equivalent solute concentration was calculated via ${\bar{C}}_{0}=\sum_{i=1}^{N}c_{0,i}$ [32], in which *N* denoted the number of solute elements. By means of the same method, the equivalent liquidus slope m, the equivalent solute partition coefficient *k*, and the equivalent solute expansion coefficient *β_c_* can be evaluated via ${\bar{C}}_{0}\cdot \bar{m}=\sum_{i=1}^{N}m_{i}c_{0,i}$, ${\bar{C}}_{0}\cdot \bar{m}/\bar{k}=\sum_{i=1}^{N}m_{i}c_{0,i}/k_{i}$, and ${\bar{C}}_{0}\cdot \left(1-\bar{k}\right)/{\bar{\beta }}_{c}=\sum_{i=1}^{N}c_{0,i}\left(1-k_{i}\right)/\beta_{ci}$ respectively. The material properties and processing parameters are listed in Table 2.

#### 4.1.2. Numerical Solution Strategy

Based on the fundamental principles of solidification theory, heat transfer, and mass transport, this study establishes a multiphysics-coupled mathematical model for the directional solidification process of castings [36,37]. The model framework integrates energy conservation equations, Navier–Stokes equations, and solute diffusion equations, incorporating energy conservation constraints and solute partitioning boundary conditions at the solid–liquid interface to achieve precise mathematical characterization of dynamic solidification front evolution. During model development, the validated multiphase volume-averaged solidification model previously established [13,14] was adopted, with detailed theoretical foundations and numerical methodologies referenced therein.

**Figure 15:**
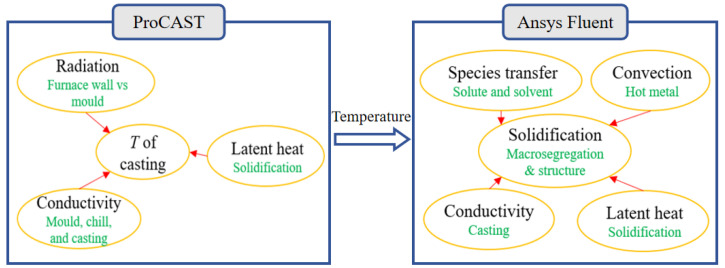
Framework of the computational strategy.

Figure 15 presents the framework of the computational strategy, which is built upon a hybrid numerical system independently developed by the research team [32]. This framework integrates three groundbreaking innovations: 

(1) Multi-software collaborative architecture: a deep coupling system between ProCAST 2019.0 (temperature field solver) and ANSYS Fluent 17.1 (flow-solute field analyzer) forms a cross-platform computational network covering multiphysical interactions.

(2) Precise latent heat treatment: an enhanced phase transition model based on the equivalent heat capacity method dynamically adjusts thermophysical parameters to accurately capture latent heat effects during solidification.

(3) Dynamic boundary mapping: an adaptive interpolation algorithm enables real-time transfer of temperature-flow boundary conditions, effectively addressing moving boundary coupling challenges.

The framework systematically investigates the influence mechanisms of convection and solidification shrinkage flow on the solute transport process by establishing a melt flow-solidification phase transformation interaction model, thereby revealing the regulatory laws of flow fields on solute segregation behavior and eutectic structure evolution.

### 4.2. Discussion and Analysis of Computational Results

#### 4.2.1. Eutectic Distribution Characteristics in the Casting

**Figure 16:**
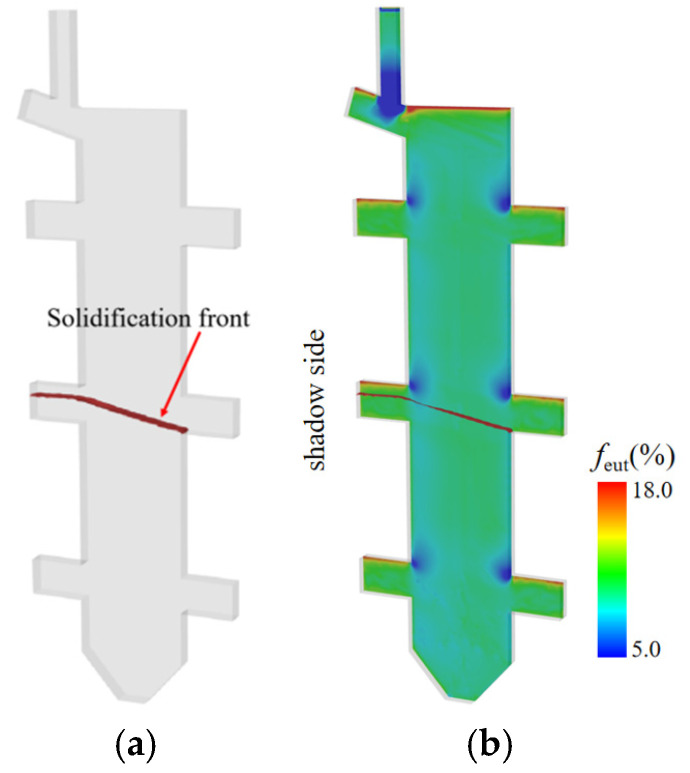
Calculated eutectic distribution in the casting: (**a**) Morphology of the solidification front at time = 2092 s; (**b**) Spatial distribution of the eutectic phase in the central cross-section after full solidification.

Figure 16 presents the numerical simulation results of the eutectic phase (f_eut_) distribution on the central cross-section of the casting. Figure 16a captures the morphology of the solidification front at time of 2092 s, while Figure 16b fully illustrates the spatial distribution pattern of the eutectic phase in the central cross-section after complete solidification. The numerical simulation results distinctly demonstrate the following features: a eutectic-rich layer with a thickness of approximately 1.5 mm is formed in the upper regions of the platform structures, and the average volume fraction of the eutectic phase (fₑᵤₜ) within this layer is significantly higher than that in other regions. With increasing casting height, enhanced eutectic accumulation occurs at elevated platform positions, suggesting a direct correlation between local solidification kinetics and eutectic phase formation. The upper region of the platform exhibits a relatively slower solidification rate, which affords ample time for the upward migration and accumulation of solutes (Al, Ti). In contrast, the lower region features a faster solidification rate, where solutes fail to accumulate adequately in time—ultimately resulting in a relatively sparse distribution of the eutectic phase.

**Figure 17:**
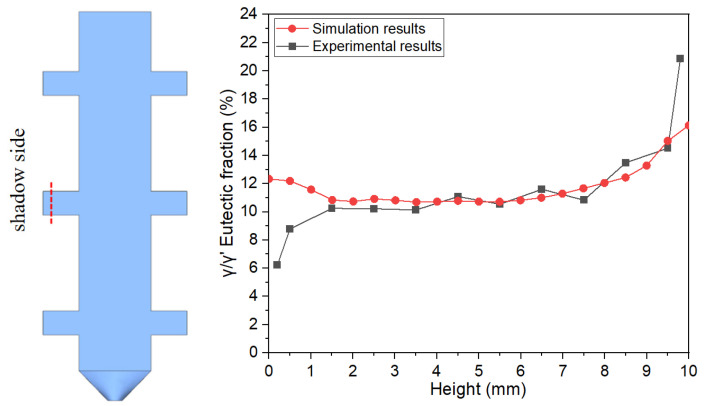
Comparison of experimental and simulated γ/γ’ eutectic content variation curves along the solidification direction at sampling locations in the 10 mm height plate casting.

**Figure 18:**
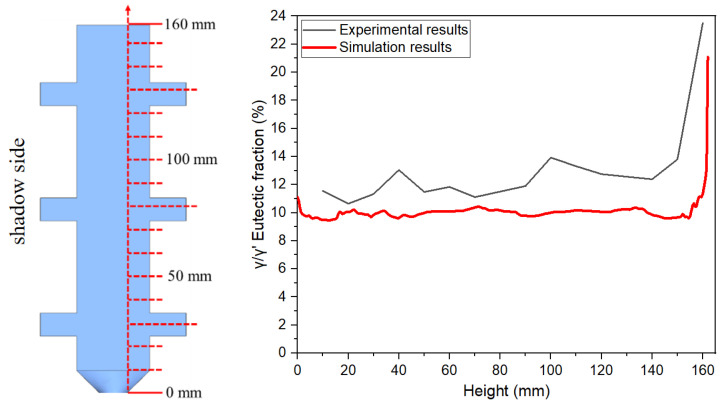
Comparison of experimental and simulated γ/γ’ eutectic content variation along the solidification direction for full-height transverse and longitudinal sampling in the 10 mm height plate casting.

Figure 17 and Figure 18 present experimental–simulation comparative results of γ/γ’ eutectic distribution at different sampling locations within the 10 mm plate. Figure 17 focuses on the directional distribution of eutectic phases at characteristic positions of the plate during solidification. Experimental measurements and simulation results exhibit strong quantitative agreement in the gradient trends of eutectic volume fraction. Figure 18 extends the analytical scope to the full height dimension of the specimen. Through systematic experimental-simulation comparisons, it is demonstrated that the numerical model can accurately reconstruct the three-dimensional spatial distribution characteristics of γ/γ’ eutectic in the casting, with prediction results showing excellent agreement with experimental measurements. Notably, the simulations not only successfully predict the spatial correspondence between eutectic-rich layers and depletion zones but also precisely capture characteristic concentration fluctuations in regions of geometric abruptness.

#### 4.2.2. Eutectic Distribution Mechanism in the Plate Region of the Casting

During the solidification process of the casting, liquid flow is primarily driven by thermosolutal buoyancy. In the unsolidified molten metal, where solute concentration remains relatively uniform, convective flow is dominated by thermal buoyancy. However, within the mushy zone near the solidification front, solute segregation occurs during phase transformation. The interdendritic liquid enriched with elements such as Al and Ti (due to their lower density) tends to rise upward, triggering solute-driven convection. This convective flow promotes the accumulation of solute-rich liquid at the upper surfaces, ultimately forming a significant volume of γ/γ’ eutectic phases upon solidification.

**Figure 19:**
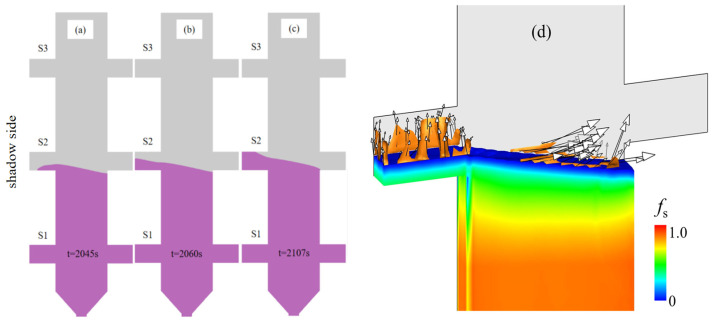
Computational results of solidification progression and solute convection phenomena on the S2 side of the 10 mm height plate: (**a**) solidification front morphology at t = 2045 s; (**b**) solidification front morphology at t = 2060 s; (**c**) solidification front morphology at t = 2107 s; (**d**) solid volume fraction *f*_s_ within boss S2 at t = 2060 s, overlaid by the iso-surface of $c_{\mathrm{mix}}=0.365$ to show the morphology of the plumes, the vectors on the plumes denote the liquid velocity.

The computational results of the solidification progress and solute convection phenomena in the boss S2 side casting are shown in Figure 19. When the solidification front advances to boss S2, the sequential solidification process is illustrated in Figure 19a–c. Prior to the arrival of the solidification front at S2, although the melt in S2 was sufficiently undercooled, the actual solidification process did not initiate immediately. Instead, it commenced only when the solidification front reached the bottom of the boss. During solidification, when the solidification front encounters geometric variations such as boss S2, significant changes occur in heat transfer and solute diffusion behaviors within the melt. At the bottom of boss S2, due to the higher undercooling of the melt, solute diffusion rates may accelerate relatively, thereby reducing the inhibitory effect of the solute boundary layer on dendritic growth. Figure 19 further presents detailed metallographic images of dendritic growth at the bottom and sidewalls of boss S2. The images clearly reveal that as dendrites progressively develop into the S2 region, their secondary dendritic arms rapidly extend toward the shadow side.

During the initial stage of solidification front advancement into the S2 region, as shown in Figure 19d, the solute convection phenomenon within boss S2 is primarily driven by thermal buoyancy effects induced by temperature gradients. As the casting solidification progresses, the enrichment of solute elements such as Al and Ti in interdendritic regions triggers localized density inversion, which drives upward solute flow within S2. In the magnified view of Figure 19d, several short-lived plumes form near the solidification front. Solute-enriched liquid is transported upward along these plumes and accumulates at the top of the platform. With continued temperature decrease, the solute-rich liquid at the platform top solidifies into a eutectic layer. These results provide an explanation for the increased eutectic volume fraction present on the top surface of each platform, as shown in Figure 8.

#### 4.2.3. Analysis of γ/γ’ Eutectic Distribution Patterns in Corner Regions

**Figure 20:**
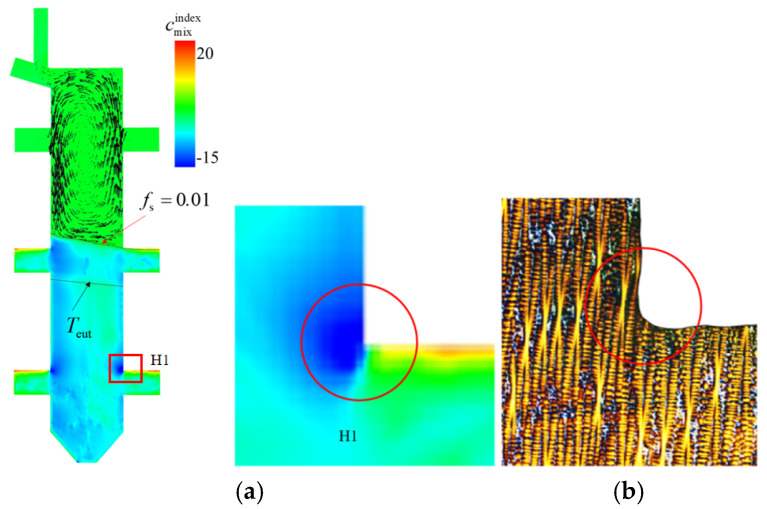
Comparison of simulated eutectic distribution and metallographic micrographs at the corner region of boss H1: (**a**) calculated mixture concentration index $c_{\mathrm{mix}}^{\mathrm{index}}$ in region H1, the black line on the top indicates the calculated solidification front of *f*_s_ = 0.01, and the black line on the bottom indicate the eutectic isotherm *T*_euc_; (**b**) metallographic image of area H1.

Figure 20 presents a comparative analysis of simulated eutectic distribution and metallographic experimental results at the corner of boss H1. During the solidification process, the interdendritic solute-enriched liquid is solidified as eutectics. The calculated mixture concentration index ($c_{\mathrm{mix}}^{\mathrm{index}}$) is shown in Figure 20a, with $c_{\mathrm{mix}}^{\mathrm{index}}=\left(c_{\mathrm{mix}}-c_{0}\right)/c_{0}*100\%$, in which $c_{\mathrm{mix}}$ is the mixture concentration and $c_{0}$ is the initial concentration. It can be seen that $c_{\mathrm{mix}}^{\mathrm{index}}$ is larger in the geometric transition zone of boss H1, which indicates a higher volume fraction of eutectic content compared to its adjacent regions. Corresponding low-magnification metallographic micrographs (Figure 20b) visually characterize the microstructural features of this area. Systematic comparison between the simulation contour plots and metallographic images revealed a marked reduction in γ/γ’ eutectic content at the boss corner compared to neighboring zones. The simulation results exhibited strong agreement with experimental observations, validating the numerical model’s capability to accurately characterize eutectic distribution features under complex geometric configurations.

**Figure 21:**
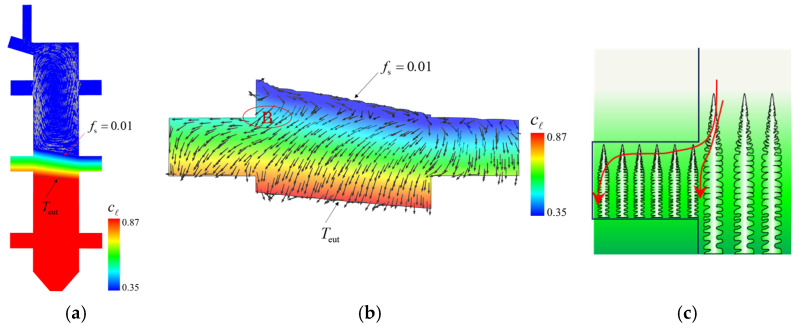
Simulation results of liquid concentration $c_{l}$: (**a**) $c_{l}$ on the vertical section of the whole casting overlaid by the liquid velocity in the bulk liquid; (**b**) $c_{l}$ in the mush zone confined between the solidification front (*f*_s_ = 0.01) and the eutectic isotherm (*T*_eut_), the vectors indicate the liquid flow in the mushy zone; (**c**) a schematic to the dendrites, liquid concentration, and the flow in the mushy zone.

The liquid concentration $c_{l}$ distribution and liquid velocity are shown in Figure 21a. Owing to the continuous rejection of solute during the solidification process, $c_{l}$ increase with the mushy zone depth. A magnified view of the liquid concentration in the mushy zone, confined between the solidification front (*f*_s_ = 0.01) and the eutectic isotherm (*T*_eut_), is displayed in Figure 21b. The liquid flow in the mushy zone is mainly driven by solidification shrinkage. To aid understanding, a schematic illustrating the dendritic structure, interdendritic liquid concentration, and liquid flow direction in the mushy zone is provided in Figure 21c. The solute-enriched interdendritic liquid near the top of the mushy zone is drawn downward to compensate for solidification-induced volume shrinkage, whereas the flow toward the top of the mushy zone carries liquid with a concentration close to the initial concentration (c_0_), leading to local solute depletion and, consequently, a lower eutectic volume fraction. At location B, the interdendritic liquid must not only feed the solidification below it but also compensate for the volume shrinkage in the protruding platform. This additional feeding results in a lower eutectic fraction at location B, as shown in Figure 20.

## 5. Conclusions

This study investigates single-crystal plate castings of a novel third-generation nickel-based superalloy using an integrated experimental and numerical simulation approach—combining ProCAST and ANSYS Fluent—to systematically explore the distribution patterns and underlying mechanisms governing the γ/γ′ eutectic phase. The principal findings are as follows:

(1) Distribution characteristics of the γ/γ′ eutectic phase

Experimental characterization performed on plate castings with boss heights of 5 mm, 10 mm, and 15 mm reveals a consistent distribution trend along the solidification direction: enrichment in the upper section, sparsity in the lower section, and uniformity in the middle section. Specifically, the upper region shows significant eutectic aggregation, with a volume fraction of 19.83–20.88%, while the lower region exhibits a markedly lower eutectic content of only 4.74–6.51%. This pattern offers essential experimental evidence for understanding the eutectic formation mechanism.

(2) Thermosolutal buoyancy-driven migration mechanism of the eutectic phase

This study systematically examined the mechanism by which liquid flow (driven by thermosolutal buoyancy) and microstructural evolution during solidification influence the distribution of γ/γ’ eutectic structures. Simulation findings reveal that thermosolutal buoyancy-driven melt convection is the primary cause of uneven eutectic distribution, specifically: during solidification, solute-enriched melt containing γ’-forming elements (e.g., Al, Ti) ascends along specific paths driven by thermosolutal buoyancy, ultimately leading to eutectic accumulation in the upper regions of the casting; in contrast, weaker fluid flow in the lower regions restricts solute aggregation, thereby yielding a sparse, dispersed distribution of the eutectic phase.

(3) Synergistic effect of multi-physical fields on eutectic microstructure

By comparative analysis of the dynamic evolution characteristics of temperature fields, solute concentration fields, and flow fields under different solidification conditions—drawing on this study’s experimental and simulation data—this research explicitly reveals the synergistic regulatory effects of these factors on both the formation and distribution of γ/γ’ eutectic structures. For example, the “temperature–concentration gradient balance” that exists in the middle region can promote the uniform distribution of eutectic structures by regulating melt flow. This study’s results further deepen the scientific understanding of superalloy solidification mechanisms, while also providing theoretical support for the subsequent optimization of superalloy casting processes.

(4) Integrated experimental–simulation research framework and its applications

An integrated methodology combining experimental characterization, multi-physics simulation, and field-overlay visualization has been established, enabling clear interpretation and validation of the γ/γ′ eutectic distribution mechanism. The results offer direct guidance for casting process optimization: by adjusting parameters such as thermosolutal buoyancy intensity and melt flow distribution, excessive eutectic aggregation can be suppressed, thereby improving microstructural homogeneity and product quality of single-crystal superalloy plate castings. This study provides theoretical and practical support for the industrial production of high-end superalloy components.

## Figures and Tables

**Table 1:** Chemical composition of the investigated superalloy (wt.%).

Element	Al	Co	Cr	Fe	Hf	Mo	Nb	Re	Ta	Ti	W	Ni
Content	5.69	5.97	3.39	0.21	0.03	0.41	0.10	4.89	8.07	0.15	6.52	Base

**Table 2:** Material properties and processing parameters [32,33,34,35].

Properties/Parameters	Symbol	Units	Values
Thermophysical			
Specific heat of the alloy	*c*_p,l_; *c*_p,s_	J·kg^−1^·K^−1^	500.0
Latent heat	Δ*h_f_*	J·kg^−1^	2.4 × 10^5^
Liquid diffusion coefficient	*D*_1_	m^2^·s^−1^	3.6 × 10^−9^
Liquid thermal conductivity	*k*_1_	W·m^−1^·K^−1^	33.5
Solid thermal conductivity	*k* *_s_*	W·m^−1^·K^−1^	24.6
Thermal expansion coefficient	*β* *_T_*	K^−1^	−1.16 × 10^−4^
Solutal expansion coefficient	*β* *_c_*	wt.%^−1^	−0.228
Density	*P*	kg·m^−3^	7646.0
Viscosity	*μ*_1_	kg·m^−1^·s^−1^	4.9 × 10^−3^
Thermodynamic			
Eutectic temperature	*T* *_eut_*	K	1627.0
Liquidus slope	*m*	K (wt.%)^−1^	−1.145
Equilibrium partition coefficient	*k*	-	0.57
Primary dendritic arm spacing	*λ* *_1_*	µm	500.0
Melting point of the solvent	*T* *_f_*	K	1728.0
Others			
Initial concentration	${\bar{C}}_{0}$	wt.%	35.09
Initial temperature	*T* *_0_*	K	1773.0
Withdrawal velocity	*v*	mm/min	3.0

## Data Availability

The original contributions presented in the study are included in the article, further inquiries can be directed to the corresponding authors.
